# Blockade of Lysosomal Acid Ceramidase Induces GluN2B-Dependent Tau Phosphorylation in Rat Hippocampal Slices

**DOI:** 10.1155/2014/196812

**Published:** 2014-09-08

**Authors:** Marie-Elaine Laurier-Laurin, Audrée De Montigny, Suzanne Attiori Essis, Michel Cyr, Guy Massicotte

**Affiliations:** Département de biologie médicale, Université du Québec à Trois-Rivières, Trois-Rivières, QC, Canada G9A 5H7

## Abstract

The lysosomal acid ceramidase, an enzyme known to limit intracellular ceramide accumulation, has been reported to be defective in neurodegenerative disorders. We show here that rat hippocampal slices, preincubated with the acid ceramidase inhibitor (ACI) d-NMAPPD, exhibit increased N-methyl-D-aspartate (NMDA) receptor-mediated field excitatory postsynaptic potentials (fEPSPs) in CA1 synapses. The ACI by itself did not interfere with either paired pulse facilitation or alpha-amino-3-hydroxy-5-methylisoxazole-4-propionate (AMPA) receptor-mediated fEPSPs, indicating that its influence on synaptic transmission is postsynaptic in origin and specific to the NMDA subtype of glutamate receptors. From a biochemical perspective, we observed that Tau phosphorylation at the Ser262 epitope was highly increased in hippocampal slices preincubated with the ACI, an effect totally prevented by the global NMDA receptor antagonist D/L(−)-2-amino-5-phosphonovaleric acid (AP-5), the calcium chelator 1,2-bis(o-aminophenoxy)ethane-N,N,N′,N′-tetraacetic acid (BAPTA), and the GluN2B (but not the GluN2A) receptor antagonist RO25-6981. On the other hand, preincubation of hippocampal slices with the compound KN-62, an inhibitor known to interfere with calcium/calmodulin-dependent protein kinase II (CaMKII), totally abolished the effect of ACI on Tau phosphorylation at Ser262 epitopes. Collectively, these results provide experimental evidence that ceramides play an important role in regulating Tau phosphorylation in the hippocampus via a mechanism dependent on GluN2B receptor subunits and CaMKII activation.

## 1. Introduction

Lipids are a heterogeneous group of molecules that are ubiquitous components of cellular membranes. Several studies over the past decades led to the concept that lipids and lipid-derived molecules are more than purely structural elements and exhibit crucial functions in signal transduction and cell regulation. For instance, in the brain, one of the most abundant classes of lipids is sphingolipids. Ceramide, which is the core structure of sphingolipids, plays an important second messenger role in a wide range of cellular functions, including proliferation, adhesion, and cell differentiation [1]. Ceramide can be formed by* de novo* synthesis, by degradation of sphingomyelin, or by reacylation of sphingoid long-chain bases. Deregulation of one of these three pathways could lead to ceramide overproduction, which has been observed in a number of neurodegeneration diseases [2–5]. In that line, increased levels of endogenous ceramide promote the biogenesis of amyloid *β*-peptide via a posttranslationally stabilization of the *β*-secretase enzyme BACE1 [6]. The abnormal accumulation of amyloid *β*-peptide into senile (or amyloid) plaques is the main pathogenic event that occurs in all forms of Alzheimer's disease. Thus, it has been suggested that ceramide elevation, A*β* formation, and Tau toxicity synergize to induce neuronal dysfunction in Alzheimer's disease [7].

The catabolism of ceramide occurs continuously in lysosomes through the activity of acid ceramidase enzyme, which catalyzes the hydrolysis of the N-acyl linkage between the sphingoid base and fatty acid of ceramide. Studies have documented that this enzyme plays important roles in limiting excessive accumulation of ceramides in cells and, in turn, avoiding the potential toxic effect of high ceramide levels. In fact, dysfunction of the human gene encoding ceramidases leads to typical lysosomal sphingolipidosis, termed Farber's disease, which is a fatal neurodegenerative condition resulting from accumulations of ceramides in lysosomes [8, 9]. The exact cascade of molecular events from ceramide accumulation to neuronal impairment in neurodegenerative diseases has not yet been clearly documented.

Ceramides have recently been implicated in membrane-trafficking events involved in the maintenance of muscarinic [10] and glutamatergic [11] receptors at the membrane surface. In particular, Wheeler et al. [12] established that enhanced ceramide levels increase the number of NMDA subtypes of ionotropic glutamate receptors in lipid rafts of hippocampal synapses. Considerable evidence suggests that NMDA receptor overactivation is important in mediating glutamatergic-induced toxicity in several neurodegenerative conditions [13]. The present project was designed to investigate how ceramide accumulation resulting from acid ceramidase inhibition may interfere with NMDA receptor function. In addition, we focused on the possibility that ceramidase inhibition may also impact the phosphorylation of Tau proteins, which is dynamically regulated by intracellular mechanisms dependent on NMDA receptor properties [14].

## 2. Materials and Methods

### 2.1. Ethics Approval

Animal care procedures were reviewed by the Institutional Animal Care Committee of Université du Québec à Trois-Rivières and were found to be in compliance with guidelines of the Canadian Council on Animal Care.

### 2.2. Pharmacological Agents

The ACI d-NMAPDD was purchased from Cayman (Ann Arbor, MI, USA). The selective GluN2A antagonist NVP-AAM077 (NVP) was a gift from Dr. Yves Auberson (Novartis Pharma AG, Basel, Switzerland). The GluN2B receptor antagonist RO25-6981 and the global NMDA receptor antagonist AP-5 were obtained from Tocris Bioscience (Ellisville, MO, USA), while the membrane-impermeable calcium chelator BAPTA was procured from BioMol (Plymouth, PA, USA). Inhibitors of protein kinase C (PKC; chelerythrine chloride), glycogen synthase kinase-3 (GSK3; SB216763), Ca^2+^/calmodulin-dependent protein kinase II (CaMKII; KN62), and protease as well as phosphatase inhibitor cocktails were supplied by Calbiochem (San Diego, CA, USA). 6-Cyano-7-nitroquinoxaline-2,3-dione disodium (CNQX) and picrotoxin were purchased from Sigma (St. Louis, MO, USA). All pharmacological agents, except NVP-AAM077 and RO25-6981, were dissolved in dimethylsulfoxide (0.05% final concentration) and mixed in artificial cerebrospinal fluid (aCSF) on the day of experimentation to obtain the desired final concentration. Both selective GluN2A and GluN2B antagonists were dissolved in water.

### 2.3. Hippocampal Slices

Male Sprague-Dawley rats (6-7 weeks of age), purchased from Charles River Laboratories (Montréal, QC, Canada), were housed for 1 week prior to any experiments in a temperature-controlled room, with free access to laboratory chow and water. For hippocampal slice preparation, the animals were anesthetized by isoflurane inhalation (Baxter Corp., Toronto, ON, Canada) and decapitated. Their brains were then rapidly removed and placed in ice-cold cutting buffer of the following composition (in mM): NaCl, 124; KCl, 3; KH_2_PO_4_, 1.25; CaCl_2_, 1; MgSO_4_, 3; NaHCO_3_, 26; glucose, 10; and saturated with carbogen (95% O_2_/5% CO_2_). Hippocampi were dissected, and transverse 350 *μ*m thick slices were prepared with a McIlwain tissue chopper. The slices were placed on a nylon mesh in a liquid-gas interface recording chamber and perfused continuously at 1.5 mL/min with preheated (34 ± 1°C) aCSF containing (in mM): NaCl, 124; KCl, 3; KH_2_PO_4_, 1.25; CaCl_2_, 3; MgSO_4_, 1; NaHCO_3_, 26 and glucose, 10, while the upper surface of the chamber was exposed to humidified carbogen. The slices were allowed to recover for at least 1 h before experimental recordings began.

### 2.4. Electrophysiology

Field excitatory postsynaptic potentials (fEPSPs) were evoked by a bipolar stimulating electrode and recorded in the CA1 stratum radiatum with a glass microelectrode containing 2 M NaCl. Stimulation consisted of a 0.1 ms pulse delivered every 30 s (0.033 Hz) with current intensity adjusted to obtain 40–50% of maximal fEPSPs. In some experiments, paired stimuli were given at various intervals (ranging from 50 to 400 ms), and paired-pulse facilitation (PPF) was determined by comparing the peak amplitude of the second relative to the first fEPSP (fEPSP_2_/fEPSP_1_ ∗ 100). In other cases, magnesium concentration in aCSF was reduced to 50 *μ*M, with 10 *μ*M CNQX and 5 *μ*M picrotoxin added to reveal the component of synaptic transmission mediated by NMDA receptors. Under these conditions, Schaffer commissural fibers were cut between areas CA1 and CA3. Field EPSPs were recorded with NAC 2.0 software (Theta Burst Corp., Irvine, CA, USA). Values are presented as means ± SEM. Drugs were prepared fresh daily in aCSF prior to being added to the recording chamber.

### 2.5. Western Blotting

After pharmacological treatment, hippocampal slices were dissected from brain sections and homogenized in ice-cold RIPA lysis buffer containing 50 mM Tris-HCl, 150 mM NaCl, 1% Triton X-100, 0.25% sodium deoxycholate, and 1 mM EDTA supplemented with protease and phosphatase inhibitor cocktails. Protein levels were measured by Bradford assay (Bio-Rad, Hercules, CA, USA). Protein lysates (40 *μ*g) were electrophoresed on 10% sodium dodecyl sulfate-polyacrylamide gel. Separated proteins were transferred onto nitrocellulose membranes, and nonspecific binding sites were blocked by incubation for 1 h at room temperature in phosphate-buffered saline, pH 7.4, containing 5% bovine serum albumin (BSA fraction V) purchased from Fisher Scientific (Pittsburgh, PA, USA).

Then, selected primary antibodies were incubated overnight at 4°C. After several washes with 0.1% Tween 20, the blots were incubated for 1 h at room temperature in specific secondary horseradish peroxidase-conjugated antibody solution. Both primary and secondary antibodies were diluted in Tris-buffered saline/0.1% Tween 20/1% BSA. Immunoreactivity was visualized by chemiluminescence reactions and densitometric scanning with Vision Work LS software (UVP Bioimaging, Upland, CA, USA); band intensity was quantified by ImageJ (W.S. Rasband, National Institutes of Health, Bethesda, MD, USA). The densitometry data were expressed as relative optical density. Labeling of *β*-actin was routinely performed to ensure identical protein loading on blots.

### 2.6. Antibodies

All antibodies reacting with Tau proteins and NMDA receptors were purchased from AbCam (Cambridge, MA, USA). The mouse polyclonal antibody Tau-5 (dilution 1 : 500) served to estimate total Tau protein levels in hippocampal extracts, along with rabbit polyclonal antibodies recognizing Tau phosphorylated at Ser199-202 (dilution 1 : 1,000), Ser262 (dilution 1 : 1,000), and Ser396 (dilution 1 : 1,000) residues. Goat anti-rabbit and goat anti-mouse peroxydase-conjugated antibodies (dilution 1: 5,000) as well as SuperSignal chemiluminescent substrate kits were procured from Pierce Chemical Co. (Rockford, IL, USA). Antibody recognizing *β*-actin (dilution 1 : 5,000) was also purchased from AbCam.

### 2.7. Statistical Analysis

Statistical significance was estimated by conventional *t*-test (electrophysiology) or analysis of variance, followed by the Newman-Keul's* post hoc* test (biochemistry). In most cases, the data are presented as means ± SEM, and values are considered to be significantly different at *P* < 0.05.

## 3. Results

### 3.1. ACI Increases NMDA Receptor-Mediated Synaptic Transmission

**Figure 1:**
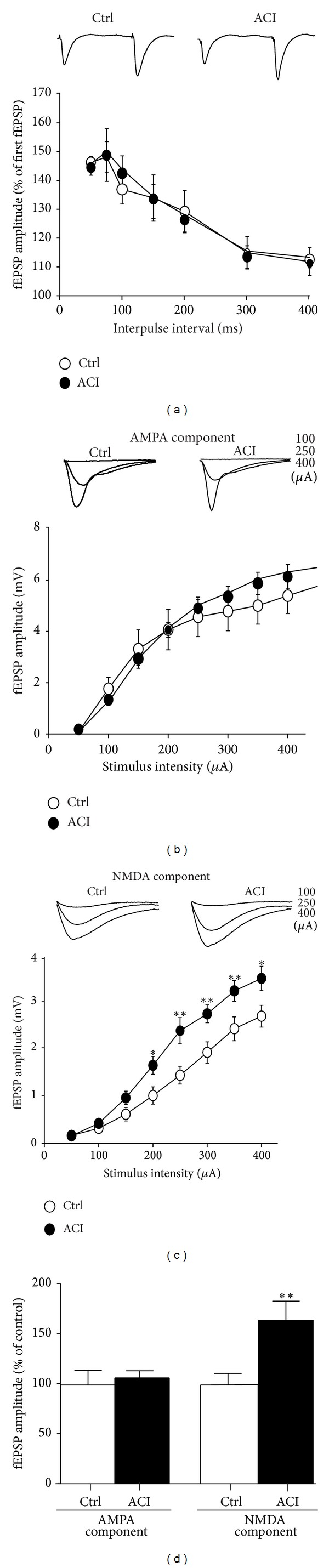
Effects of d-NMAPPD on PPF and AMPA- and NMDA-mediated components of synaptic transmission. Hippocampal slices were maintained in normal aCSF containing 25 *μ*M d-NMAPPD for at least 3 h prior to and during recording. (a) PPF of fEPSPs was measured at numerous interpulse intervals ranging from 50 to 400 ms in control and d-NMAPPD-treated slices. Representative traces are shown for the 50 ms interpulse interval. Data were determined by comparing the peak amplitude of the second relative with the first fEPSP (fEPSP_2_/fEPSP_1_ ∗ 100) and are means ± SEM obtained from 8 different rats (3 measurements per slice). (b) Graph illustrating AMPA-mediated components of synaptic transmission recorded at different stimulus intensities (50 to 400 *μ*A) in a normal aCSF. Representative traces are shown for the 100, 250, and 400 *μ*A stimulus intensities. Data on the amplitude of fEPSPs recorded in control slices and slices preexposed to d-NMAPPD (3 h) are presented in the bar graph (means ± SEM of 3 measurements per slice) obtained from 8 different rats. No statistical differences were observed between d-NMAPPD versus control slices (*t*-test). (c) As in B, but NMDA-mediated components were pharmacologically isolated in a modified aCSF containing a low-magnesium concentration as well as antagonists for both AMPA and GABA_A_ receptors (see Methods). In these conditions, Schaffer-commissural fibers were also cut between areas CA1 and CA3 sectors to reduce spiking in pyramidal cells. Data relative to NMDA-mediated components of synaptic transmission recorded in control slices and slices preexposed to d-NMAPPD (3 h) are presented in the bar graph (means ± SEM of 3 measurements per slice) obtained from 8 different rats. *P < 0.05, **P < 0.01, d-NMAPPD versus control values (*t*-test). (d) Summary data on the amplitude of fEPSPs recorded at a stimulus intensity of 250 *μ*A are expressed in percentage of control values and presented in the bar graph (means ± SEM of 3 measurements) obtained from 8 different rats. Note the ability of d-NMAPPD to enhance specifically the NMDA-mediated component of synaptic transmission. **P < 0.01, d-NMAPPD versus control (*t*-test).

Electrophysiological experiments were performed to determine whether acid ceramidase blockade alters synaptic transmission in CA1 pyramidal cells. Towards this end,* in vitro* hippocampal slices were prepared and perfused with aCSF containing the cell-permeable ACI, d-NMAPPD, before delivering electrical stimulation to Schaffer collateral inputs in area CA1 of the hippocampus. Our experiments were conducted in slices preincubated for at least 3 h with 25 *μ*M d-NMAPPD, conditions that have been reported to induce massive increases of ceramide levels in numerous cell types [15]. In a first series of experiments, standard assays of changes in transmitter release were undertaken by measuring the degree of PPF. In that line, 2 stimulation pulses were delivered to Schaffer commissural fibers at several interstimulus intervals (50, 75, 100, 150, 200, 300, and 400 ms). As illustrated in Figure 1(a), we observed that, for each interstimulus interval, no substantial changes occurred in mean facilitation of the second responses between control and d-NMAPPD-treated slices. Of course, this constitutes strong evidence that disturbance in transmitter release is probably not associated with acid ceramidase inhibition.

In these slices, we conducted additional recordings in which we analyzed the possibility that acid ceramidase inhibition might interfere with the postsynaptic components of synaptic transmission in CA1 pyramidal cells of the hippocampus. First, routine synaptic transmission, which is essentially driven by AMPA receptors in CA1 neurons [16], was monitored at different stimulus intensities. No substantial modification in amplitude of AMPA-mediated components was observed between control and d-NMAPPD-treated slices when stimulus intensity was increased from 50 to 400 *μ*A (Figure 1(b)).

In another set of experiments, NMDA-mediated components of synaptic transmission were isolated pharmacologically in aCSF containing a low-magnesium concentration (50 *μ*M), 10 *μ*M of the AMPA receptor antagonist CNQX, and 5 *μ*M picrotoxin; under these conditions, Schaffer commissural fibers were also cut between areas CA1 and CA3 to avoid spiking activities. NMDA-mediated responses were then generated at various stimulus intensities in area CA1 of the hippocampus and presented as the mean amplitude of evoked fEPSPs. As reported in Figure 1(c), d-NMAPPD introduction in the slice chamber provoked pronounced increase amplitude of the NMDA component. At most stimulus intensities tested (50 to 400 *μ*A), the amplitude of NMDA receptor responses was enhanced by more than 60% in d-NMAPPD-treated slices (25 *μ*M, 3 h), relative to values estimated in control slices.

**Figure 2:**
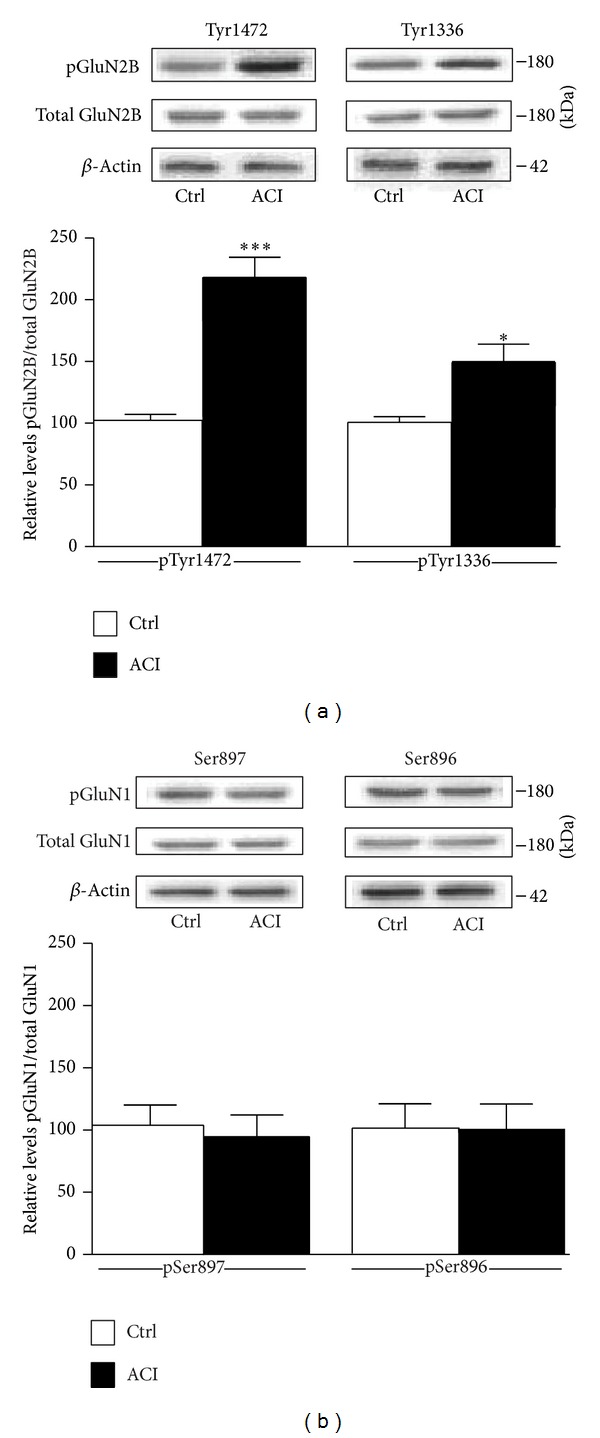
D-NMAPPD treatment is associated with increased phosphorylation of GluN2B receptor subunits. Levels of  total or phosphorylated NMDA receptor subunits were estimated by Western blotting on cell extracts obtained from hippocampal slices treated with d-NMAPPD (25 *μ*M) for 3 h. (a) Phosphorylated GluN2B levels at Tyr1472 and Tyr1336 epitopes were expressed relative to total GluN2B subunit levels. (b) Phosphorylated GluN1 levels at Ser896 and Ser897 epitopes were expressed relative to total GluN1 subunit levels. Summary data are shown in the bar graph (means ± SEM of 6 different experiments). Since these experiments were performed independently, statistical significance was estimated by conventional unpaired *t*-test. *P < 0.05, ***P < 0.001, and d-NMAPPD (3 h) versus the controls. Note the ability of d-NMAPPD to strongly enhance GluN2B receptor phosphorylation at residue Tyr1472.

We concluded, from these electrophysiological experiments, that the influence of acid ceramidase inhibition on synaptic transmission was postsynaptic in origin and specific to the NMDA subtype of glutamate receptors (see Figure 1(d)). In line with this notion, we found that ACI treatments were associated with significant enhancement of Tyr1472 phosphorylation of GluN2B subunits and, to a lesser degree, with GluN2B-Tyr1336 phosphorylation (Figure 2(a)). However, the effect was rather specific, as d-NMAPPD did not appear to accentuate phosphorylation of other epitopes controlling NMDA receptor function, such as GluN1-Ser896 and -Ser897 (Figure 2(b)).

### 3.2. ACI Accentuates Tau Phosphorylation at the Ser262 Epitope

**Figure 3:**
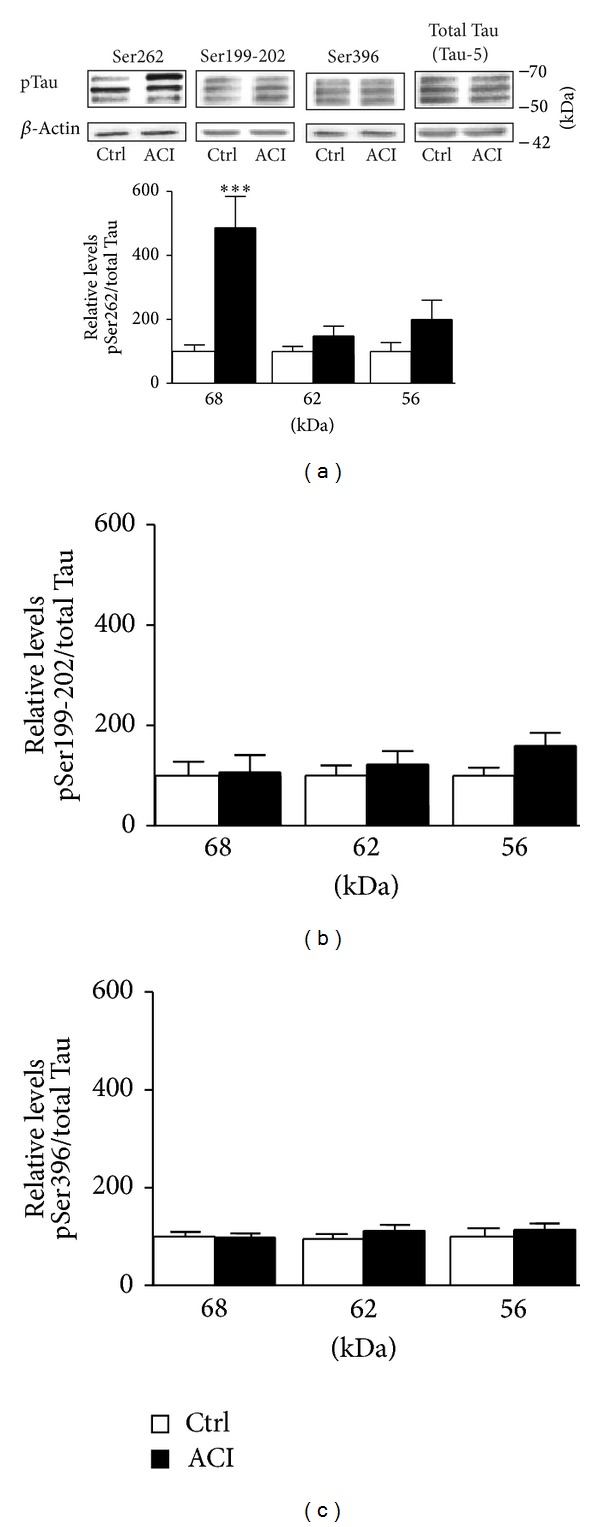
D-NMAPPD treatment promotes Tau hyperphosphorylation at the Ser262 epitope. Phosphorylated Tau levels were estimated by Western blot on cell extracts obtained from hippocampal slices treated or not with the acid ceramidase inhibitor d-NMAPPD (25 *μ*M, 3 h). Three Tau isoforms were consistently distinguished in immunoblots performed with antibodies directed against various phosphoepitopes (Ser262, Ser199-202, and Ser396). Summary data on pSer262 (a), pSer199-202 (b), and pSer396 (c) epitopes are expressed relative to total Tau (Tau-5) and shown in the bar graph (means ± SEM of 6 different experiments). For statistical analysis, one-way ANOVA was followed by Newman-Keul's* post hoc* test. ***P < 0.001, d-NMAPPD versus the controls. Note the strong ability of d-NMAPPD to enhance Tau phosphorylation at residue Ser262 of the 68 kDa isoform.

NMDA receptors have been considered to play important roles in the pathogenesis of several brain diseases implicating, for instance, Tau dysfunctions. Consequently, we explored the possibility that d-NMAPPD-induced upregulation of NMDA responses might impact Tau phosphorylation at different epitopes in hippocampal slices kept metabolically active in oxygenated aCSF. Hippocampal slices from rats were first preincubated for 3 h with d-NMAPPD, and Tau phosphorylation was then processed according to Western blotting procedures. In initial experiments, we observed that Ser262 residues, located in the microtubule-binding domain of Tau proteins, became hyperphosphorylated when exposed to the ACI. Interestingly, a consistent feature in our experiments was the preferential modulation of Tau isoforms by d-NMAPPD, as depicted in Figure 3. Lysosomal acid ceramidase inhibition, in obvious contrast with 68 kDa Tau isoforms, produced no reliable changes in phosphorylation for the other two isoforms detected and estimated, with the help of molecular weight standards, to be around 62 and 56 kDa (Figure 3(a)).

Tau has been found to possess more than 84 different phosphorylation sites [17–19], and we tested whether d-NMAPPD treatment also affects other phosphorylated epitopes.  Figure 3(b) shows that preincubation of hippocampal slices with 25 *μ*M d-NMAPPD for 3 h failed to elicit changes in phosphorylation at Ser199-202 residues, a phosphorylation site positioned in the proline-rich domain of Tau proteins. Similarly, Western blotting experiments indicated that phosphorylation of an epitope located in the C-terminal domain of Tau, Ser396, was not substantially increased by the ACI (Figure 3(c)).

### 3.3. Tau Hyperphosphorylation Is Mediated by Calcium and GluN2B Receptor Activation

**Figure 4:**
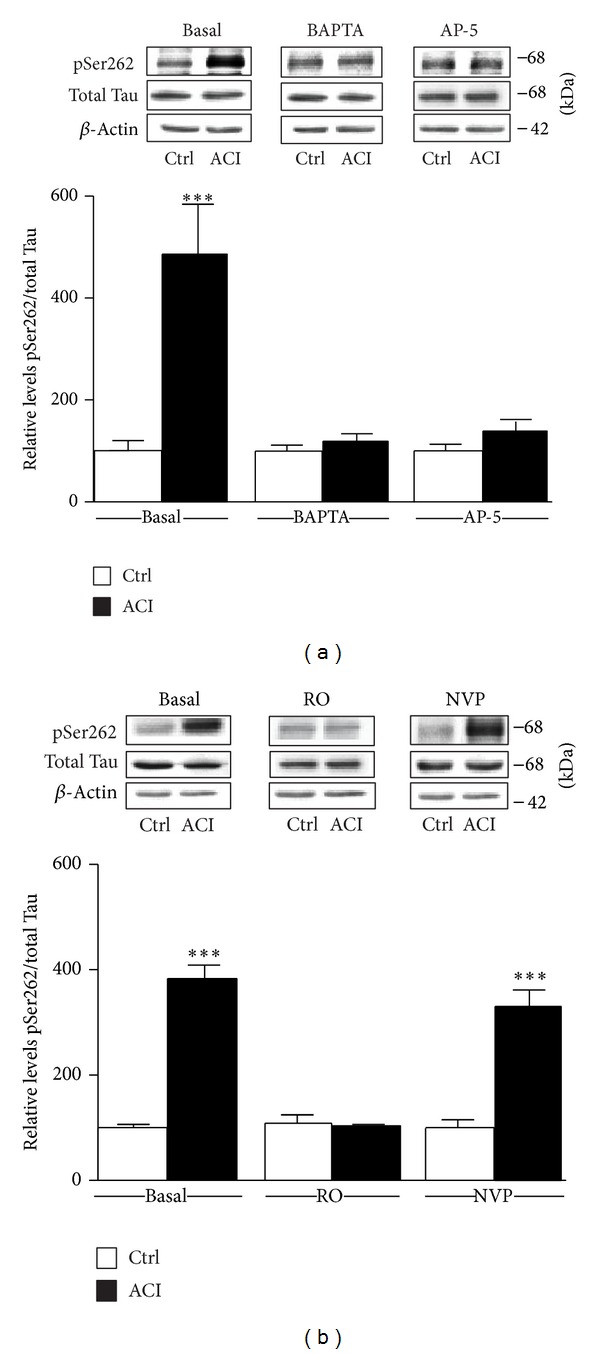
Tau hyperphosphorylation is mediated by calcium and is dependent on GluN2B receptor activation. Phosphorylated Tau levels at Ser262 were estimated by Western blotting of cell extracts obtained from hippocampal slices. In basal conditions, slices were only exposed to the acid ceramidase inhibitor d-NMAPPD (25 *μ*M, 3 h). In parallel experiments, d-NMAPPD-treated slices were preincubated (a) with the nonpermeable calcium chelator BAPTA (10 *μ*M) or with the global NMDA receptor antagonist AP-5 (50 *μ*M) and (b) with the GluN2B receptor antagonist RO25-6981 (1 *μ*M) or the GluN2A receptor antagonist NVP-AAM077 (50 nM). Summary data are expressed relative to total Tau (Tau-5) levels and shown in the bar graph (means ± SEM of 6 different experiments). For statistical analysis, one-way ANOVA was followed by Newman-Keul's* post hoc* test. ***P < 0.001, drug treated versus controls.

Tau phosphorylation appears to be enhanced in several conditions through activation of calcium-dependent pathways [14, 20]. Experiments were, therefore, conducted to determine whether the ability of d-NMAPPD to engage Tau hyperphosphorylation is reduced by calcium chelation. The data presented in Figure 4(a) indicate that d-NMAPPD treatment failed to induce Tau hyperphosphorylation in slices exposed to the calcium chelator BAPTA (10 *μ*M). Interestingly, we also observed that preexposure of hippocampal slices to AP-5 (50 *μ*M) completely blocked d-NMAPPD-induced Tau phosphorylation at Ser262 epitopes (Figure 4), indicating that Tau hyperphosphorylation mainly relies on calcium influx and NMDA receptors.

Hippocampal synapses mainly contain two types of NMDA receptors, namely, NMDA receptor subtype 2A (GluN2A) and GluN2B. Here, selective antagonists were tested to study whether d-NMAPPD-induced Tau phosphorylation requires GluN2A or GluN2B receptor activation. The possibility that stimulation of GluN2B-containing NMDA receptors is responsible for upregulating Tau phosphorylation was considered first.  Figure 4(b) illustrates that the ability of NMDA to induce Tau phosphorylation was totally abrogated in slices preexposed to the GluN2B antagonist RO25-6981 (1 *μ*M). In contrast, we noted that blockade of GluN2A-containing receptors with NVP-AAM077 (50 nM) failed to interfere with the capacity of d-NMAPPD treatments to upregulate Tau phosphorylation of Ser262 epitopes. These data are consistent with the notion that inhibition of acid ceramidase may induce Tau hyperphosphorylation by activating GluN2B-containing NMDA receptors.

### 3.4. Involvement of CaMKII

**Figure 5:**
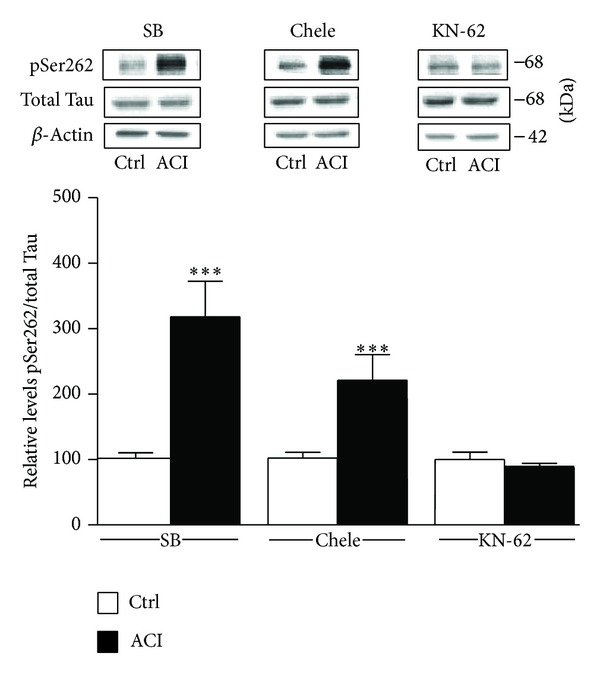
Tau hyperphosphorylation is mediated by the CaMKII pathway. As in Figure 4, except that the GSK3 inhibitor SB216763 (SB; 10 *μ*M), the PKC inhibitor chelerythrine chloride (Chele; 1 *μ*M), and the CaMKII inhibitor (KN-62; 1 *μ*M) were employed. Summary data are expressed relative to total Tau (Tau5) levels and shown in the bar graph (means ± SEM of 6 different experiments). For statistical analysis, one-way ANOVA was followed by Newman-Keul's* post hoc* test. ***P < 0.01, drug-treated versus the controls.

We finally turned our attention to the potential mechanisms underlying Tau hyperphosphorylation at Ser262 epitopes. Given our results that d-NMAPPD-induced Tau phosphorylation seems to be dependent on GluN2B receptor activation and calcium mobilization, we next investigated whether ACI can exert its action via intracellular pathways known to be regulated by calcium ions, namely, GSK3, PKC, and CaMKII pathways. In these experiments, the inhibitors were applied 30 min prior to d-NMAPPD exposure to ensure optimal enzymatic inhibition.  Figure 5 shows that the ability of d-NMAPPD to upregulate Tau phosphorylation at Ser262 residues was not substantially affected by the GSK3*β* inhibitor SB216763 (10 *μ*M) or by the PKC inhibitor chelerythrine chloride (10 *μ*M). Interestingly, however, we noticed that regulation of Tau phosphorylation during lysosomal acid ceramidase inhibition was totally abrogated in slices pretreated with the CaMKII inhibitor KN-62 (10 *μ*M).

## 4. Discussion

In recent years, it has been proposed that ceramides exert broad biological functions in cells, ranging from control of membrane receptors to the generation of signalling molecules affecting cell viability [5, 21–23]. The present study identifies lysosomal acid ceramidase, an enzyme known to limit excessive ceramide accumulation in cells [24], as an important pathway regulating the NMDA type of glutamate receptors. We observed that disruption of ceramidase activity causes a selective rise in NMDA-mediated responses in hippocampal slices, likely resulting from higher levels of GluN2B receptor activity, which probably triggers Tau hyperphosphorylation via a CaMKII pathway.

Previous investigations have examined the potential role of ceramides in the regulation of membrane receptors. For instance, ceramide production has been proposed to be involved CD40, CD95, and Fc-gamma receptor clustering [25–27]. Consistent with their potential participation in the regulation of neurotransmitter systems, initial reports examining the role of ceramides in the control of acetylcholine receptors indicated that these sphingolipids are possibly involved in controlling cell-surface expression of both nicotinic [10, 28, 29] and muscarinic [30] subtypes of receptors [10, 28, 29]. The present study, in conjunction with previous findings [12], indicates that enzymatically accumulated ceramides also interact with mechanisms capable of favoring the upregulation of NMDA-mediated responses.

Our data suggest that ceramide accumulation, resulting from lysosomal acid ceramidase inhibition, can enhance NMDA receptor activity via a mechanism involving initially GluN2B receptor phosphorylation. We demonstrated precisely that, after lysosomal acid ceramidase inhibition, phosphorylation of GluN2B-Tyr1472 was accentuated in rat hippocampal slices, whereas the same treatment was slightly effective in regulating the GluN2B-Tyr1336 epitope and totally ineffective in modulating residues specific to GluN1 subunits (Ser896 and Ser897). Several studies have shown that PKC and PKA act on GluN1-Ser896 and -Ser897, respectively, to accentuate NMDA receptor function [31–33]. Our observation that lysosomal acid ceramidase inhibition has no effect on GluN1 residues suggests that these kinase pathways are not involved. Of course, additional experiments are required to determine the mechanism underlying the preferential regulation of GluN2B-Tyr1472 epitope by ACI. By activating Src family kinases, such as Src and Fyn, ceramides might have the potential to accentuate GluN2B-Tyr1472 phosphorylation [34]. Interestingly, Fyn-mediated phosphorylation of this epitope is known to block NMDA receptor endocytosis in neurons, and one actual hypothesis is that inhibition of lysosomal acid ceramidase activity potentiates NMDA-mediated synaptic transmission by activating this pathway [33, 35].

Alternatively, studies have revealed that GluN2B-Tyr1472 is specifically dephosphorylated by the protein phosphatase STEP (Striatal-Enriched Protein Tyrosine Phosphatase) [36, 37]. It would be particularly interesting to determine whether STEP inactivation might eventually account for GluN2B-Tyr1472 phosphorylation and potentiation of NMDA-mediated responses after lysosomal acid ceramidase inhibition. On the other hand, it is well known that ceramides are precursor molecules of derivatives capable of influencing cell functions. For instance, intracellular accumulation of active metabolites, such as sphingosine and sphingosine-1-phosphate, is able to modulate numerous cellular events, such as apoptosis and cell proliferation [38, 39]. In this respect, further studies are required to determine whether ceramide derivatives are inclined to modulate GluN2B-Tyr1472 phosphorylation in hippocampal slices, and, if so, what role these derivatives play in the regulation of Tau phosphorylation.

Additional evidence on the importance of lysosomal acid ceramidase activity in controlling NMDA receptor functions comes from the investigation of basal transmission in pyramidal neurons. Application of the ACI produces a substantial increase in pharmacologically isolated NMDA-mediated responses with no apparent change in AMPA-mediated transmission at CA1 synapses. Although the precise mechanism of increased NMDA receptor activity remains unknown, high ceramide levels would be expected to alter development of NMDA-dependent forms of synaptic plasticity. Accordingly, exogenously applied C6 ceramide was previously found to prevent the formation of long-term potentiation (LTP) in CA1 pyramidal cells of the hippocampus (a synaptic model of learning and memory) [40], suggesting that high ceramide levels are detrimental on synaptic plasticity. However, it is not clear at this time if this electrophysiological defect is due to the action of ceramides on expression mechanisms of LTP involving, for instance, regulation of AMPA subtype of glutamate receptors [41]. Added to the complexity of this question are data showing that ceramide production via the neutral sphingomyelinase-2 (nSMase2) may actually favor LTP formation in the hippocampus as well as spatial memory in mice [12].

According to Wheeler and his group, rapid versus long term exposure to ceramides may have differential consequences for the development of synaptic plasticity. On that line, they proposed that a prolonged increase in the ceramide content of neuronal membranes may perturb NMDA receptor trafficking and could increase the susceptibility of neurons to excitotoxic death by locking NMDA receptors at the plasma membrane for prolonged periods of time [12]. A common mechanism that might support the development of various pathologicals conditions such as Alzheimer's disease and HIV-associated dementia [42, 43]. Interestingly, recent investigations have shown that basic alterations in cell metabolism selectively enhance NMDA receptor activity and engage a slow form of excitotoxicity over time [44]. This contention is supported by our biochemical experiments revealing that the increased NMDA receptor activity, observed after lysosomal acid ceramidase inhibition, leads to Tau hyperphosphorylation in a GluN2B-dependent manner. From a signaling perspective, the consequent increase in Tau phosphorylation appears to involve calcium mobilization and preferentially implies phosphorylation of Tau-Ser262 by the CaMKII pathway. These results are indeed in agreement with other experiments showing that activated CaMKII preferentially targets Ser262 of Tau [45].

## 5. Conclusion

**Figure 6:**
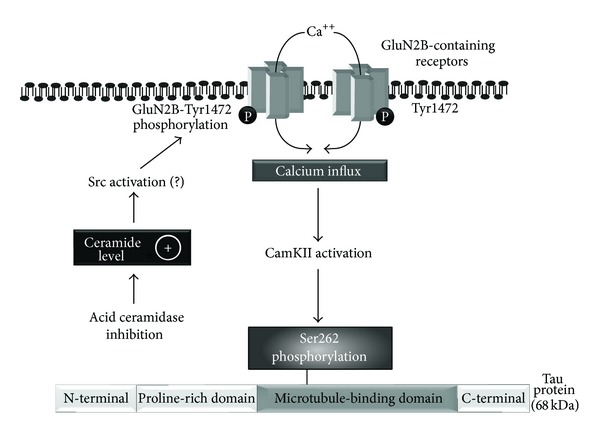
Model of Tau phosphorylation during acid ceramidase inhibition. Endogenous accumulation of ceramide is known to be rapidly observed during acid ceramidase inhibition by d-NMAPPD. Here, this inhibitor appears to accentuate NMDA receptor function through a mechanism involving GluN2B receptor phosphorylation at the Tyr1472 epitope. Consequently, calcium might selectively enhance the phosphorylation of Ser262 residues in the microtubule-binding domain of Tau via activation of the CaMKII pathway. Physiologically, acid ceramidase inactivation may induce Tau hyperphosphorylation which could have an impact on protein localization and aggregation in cells and, ultimately, on neuronal survival.

In summary, our data point to the following model: lysosomal acid ceramidase inhibition leads to ceramide accumulation, resulting in GluN2B receptor phosphorylation and selective enhancement of NMDA-mediated responses in CA1 synapses, an event that could eventually increase Tau phosphorylation by a CaMKII-dependent pathway (Figure 6). We envision that, during long-term exposure to ceramides, hippocampal neurons could eventually become more sensitive to excitotoxic damage, a scenario that will require further exploration. In view of the emerging involvement of ceramides in neurodegenerative disorders, such as Farber's disease [24] and Alzheimer's disease [43], our observations support the notion that prevention of these lipids formation might provide potential novel therapeutic approaches promoting neuronal survival [46]. However, evaluation of ceramide levels within hippocampal slices was not performed in the course of the present study. In fact, additional investigations will be necessary to examine whether specific ceramide species or their cellular location, in particular in lipid-raft domains, are mechanistically linked with the observed effects. Along this line, we recently performed* in vitro* experiments to test the influence of exogenously-applied ceramides on different phosphorylation sites of GluN subunits. Our preliminary observation is that exposure of rat hippocampal slices to the short-chain cell-permeable C2-ceramide could accentuate phosphorylation of GluN2B receptor subunits at the Tyr1472 epitope (data not shown). A finding which strongly supports the present contention that d-NMAPPD might indeed regulate NMDA receptor properties by enhancing endogenous production of ceramides. On the other hand, such a change in receptor properties by ceramides and/or their metabolic derivatives could theoretically favour the appearance of adverse neuropathological effects in hippocampal subregions other than CA1 and should be evaluated further.
